# Mr. George Combe

**Published:** 1858-11

**Authors:** 


### NECROLOGICAL NOTICES.

GEORGE COMBE.—In the death of this eminent man, which took place on the 14th of August, 1858, while on a visit to a friend near London, the cause of mental progress and improvement has lost one of its most strenuous and able advocates and promoters. For the last forty years his name has been intimately and approvingly connected with a system of mental philosophy known as the phrenological, of which he must be regarded in the light of the most logical and sagacious expounder. Mr. Combe was bred to the law, and became a writer to the *Signet,* which answers very nearly to the title of attorney in England. He continued to practice in his profession down to the year 1838; but his first appearance as an author, and at the same time an indication of the field of inquiry on which he had fully entered, were manifested in the publication of his "Essays on Phrenology." This work at once established his reputation as an able dialectician, even among those who were backward in joining him in his conclusions. Henceforth he devoted his chief energies and a large share of his time to the elucidation of the new system of intellectual and moral philosophy, and to a demonstration of its varied and important bearings on education, social polity and jurisprudence. We cannot in these few lines, meant to commemorate the worth of the man, enter into details of his labors for bettering the condition of his fellow creatures, by his pointing out the many evils which result from a neglect of natural laws, and the ameliorations which necessarily accompany a return to these laws. Nor can we give, at this time, even the titles of his numerous library productions. Of one of them special mention must, however, always be made: we refer to "The Constitution of Man Considered in Relation to External Objects," which has had a circulation in Great Britain and the United States equaled by few books of any description, and has been translated into most of the languages of Europe. Mr. Combe wrote and labored for the masses, for the people in the most comprehensive sense: his reforms were not urged in any factious spirit or for partial application, but in a calm, reasoning and practical manner, adapted both to the comprehension and the wants of those to whom he addressed himself.

Mr. Combe visited this country in the latter part of the year 1838, and remained in it nearly two years. During this period he delivered, in different parts of the United States, lectures to the number of a hundred and fifty-eight on Phrenology. His mastery of the subject, and the new and striking lights in which he presented it, although deriving little aid from the powers of elocution or grace of manner, made these lectures singularly attractive to audiences of the highest education and character in the country. His classes in Philadelphia were larger than those in any other city, on either side of the Atlantic. On his return to Edinburgh, his native city, and always the place of his fixed residence, Mr. Combe published the result of his observations and inquiries during his trans-Atlantic travels, in a work entitled "Notes on the United States of North America, during a Phrenological Visit in 1838–9–40," which contains many apposite details relating to our public improvements, schools, and social and political condition, accompanied with remarks and suggestions, indicating the shrewdness and common sense of a practical philosopher.

Mr. Combe was married in 1833 to a daughter of the celebrated Mrs. Siddons. This lady accompanied him in his travels in this country, and attended him in the last moments of a life, the married portion of which he used to say seemed to him like a happy day. Mr. Combe had reached the seventieth year of his age at the time of his death.

